# Generalized Reciprocity in Rats

**DOI:** 10.1371/journal.pbio.0050196

**Published:** 2007-07-03

**Authors:** Claudia Rutte, Michael Taborsky

**Affiliations:** Department of Behavioral Ecology, Institute of Zoology, University of Berne, Berne, Switzerland; University of Lausanne, Switzerland

## Abstract

The evolution of cooperation among nonrelatives has been explained by direct, indirect, and strong reciprocity. Animals should base the decision to help others on expected future help, which they may judge from past behavior of their partner. Although many examples of cooperative behavior exist in nature where reciprocity may be involved, experimental evidence for strategies predicted by direct reciprocity models remains controversial; and indirect and strong reciprocity have been found only in humans so far. Here we show experimentally that cooperative behavior of female rats is influenced by prior receipt of help, irrespective of the identity of the partner. Rats that were trained in an instrumental cooperative task (pulling a stick in order to produce food for a partner) pulled more often for an unknown partner after they were helped than if they had not received help before. This alternative mechanism, called generalized reciprocity, requires no specific knowledge about the partner and may promote the evolution of cooperation among unfamiliar nonrelatives.

## Author Summary

The evolution of cooperation is based on four general mechanisms: mutualism, where an action benefits all partners directly; kin selection, where related individuals are supported; “green beard” altruism that is based on a genetic correlation between altruism genes and respective markers; and reciprocal altruism, where helpful acts are contingent upon the likelihood of getting help in return. The latter mechanism is intriguing because it is prone to exploitation. In theory, reciprocal altruism may evolve by direct, indirect, “strong,” and generalized reciprocity. Apart from direct reciprocity, where individuals base their behavior towards a partner on that partner's previous behavior towards themselves, and which works under only highly restrictive conditions, no other mechanism for reciprocity has been demonstrated among conspecifics in nonhuman animals. Here, we tested the propensity of wild-type Norway rats to help unknown conspecifics in response to help received from other unknown partners in an instrumental cooperative task. Anonymous receipt of help increased their propensity to help by more than 20%, revealing that nonhuman animals may indeed show generalized reciprocity. This mechanism causes altruistic behavior by previous social experience irrespective of partner identity. Generalized reciprocity is hence much simpler and therefore more likely to be important in nature than other reciprocity mechanisms.

## Abstract 3

Empirical evidence from rats supports the theory of generalized reciprocity, in which individuals are more likely to cooperate with an unknown individual if they have received help in the past.

## Introduction

Cooperation among unrelated individuals may be achieved by reciprocal altruism in which two or more individuals help each other in turn [1,2]. The decision to cooperate is based on expected future help, which may be judged from past interactions. Most theoretical models of reciprocal altruism assume that individuals base their behavior on knowledge about a partner's previous behavior, either towards themselves (direct reciprocity [3]) or towards others (indirect reciprocity [4–6]). According to direct reciprocity, A helps B because B has helped A before; individuals remember who did what in past interactions with them and base their decision whether to cooperate or defect on this knowledge [3]. According to indirect reciprocity, A helps B because B has helped C before; indirect reciprocity involves reputation, which increases through helping and is assessed to decide whether to help a partner or not [4–6]. Both direct and indirect reciprocity require that animals possess specific cognitive abilities [7], which may impede the evolution of cooperation through these mechanisms. Strong reciprocity assumes that individuals punish noncooperators altruistically [8–10]. So far, experimental evidence for strategies predicted by direct reciprocity models remains controversial [11–16], and indirect and strong reciprocity have been found only in humans so far [8,17] (but see [18] for a possible example at the interspecific level in a cleaner fish mutualism).

Recent theoretical models have shown that cooperation could evolve even without individual recognition in small groups when individuals base their decision on the outcome of previous interactions with anonymous partners [19,20]. This mechanism, called generalized reciprocity (also “upstream tit-for-tat,” or “upstream indirect reciprocity” [21,22]), leads to cooperation because previous interactions provide information about the overall level of cooperation within the group. For instance, if it pays more to cooperate in a cooperative environment than in a noncooperative one, generalized reciprocity may establish stable levels of cooperation when the decision to stay or leave a group evolves simultaneously with the decision to cooperate [23]. The selective force promoting cooperation in generalized reciprocity is thus of the type Lehmann and Keller [24] classified as “repeated interactions with direct or indirect information on the behavior of the partner in previous moves.” However, it is important to note that under generalized reciprocity, individual recognition and specific social memory are not required, hence possibly this represents a more general mechanism leading to cooperation in animals than direct and indirect reciprocity, which require cognitive abilities potentially impeding their operation in animals [16,25]. Simple decision rules such as “walk away when encountering noncooperation” may suffice to stabilize cooperation [26].

Generalized reciprocity has been shown in humans; prior receipt of help increased the propensity to help a stranger [27–29]. A typical situation to show that past positive experience increases the future helpfulness of subjects towards unknown persons is that people who found a coin in the coin return of a public telephone were more likely to help a stranger pick up papers that had been dropped than control subjects [30]. To know whether such behavior is caused by cultural experience or shaped by natural selection, it is important to study whether similar reactions to anonymous experience can be found in nonhuman animals, which would clearly hint that an evolutionary mechanism is involved. For generalized reciprocity, special cognitive abilities are not required, as individuals only need to remember and act upon their own last experience with any partner. This may work with the help of rather simple hormonal or neuronal mechanisms triggering the propensity to cooperate. Yet, so far, to our knowledge, no experimental study has investigated the influence of anonymous prior experience on cooperative behavior in nonhuman animals.

**Figure 1:**
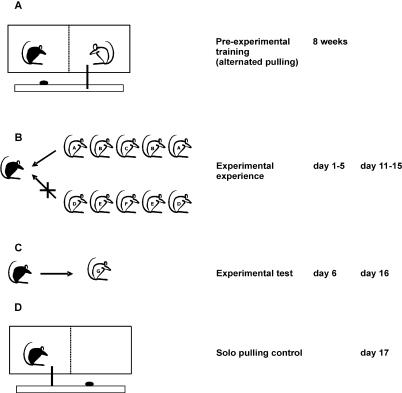
Rats Cooperate in an Instrumental Task (A) Experimental set-up: two rats in the test cage are separated by a wire mesh. By pulling a stick fixed to a baited tray one rat produces food (an oat flake) for the partner rat but is not rewarded herself for this behavior. (B) Experience phase: the focal rat (black) either experienced help by three different partner rats (A, B, or C) that pulled (test treatment; help is indicated by an arrow), or it experienced no help by three different partner rats (D, E, or F) that did not pull (control treatment; no help is indicated by a crossed arrow). (C) Test phase: the pulling behavior of the focal rat was tested against a new partner (rat G). (D) Solo pulling control: the pulling behavior of the focal rats was tested when alone in the cage. The time schedule of the experiment is also shown. Each focal rat was exposed to both test and control treatments in a randomized sequence. There was a four-day interval between the first experimental test (day 6) and the start of the second experience phase (on day 11).

Here, we studied whether cooperative behavior in rats is influenced by social experience, irrespective of the identity of partners. First, we trained female wild-type rats *(Rattus norvegicus)* in an instrumental cooperative task; by pulling a stick fixed to a baited tray a rat produced food for its partner but not for herself (Figure 1A). Second, we manipulated the focal rat's experience to receive help from a series of unfamiliar partners. The focal rat either experienced help by three different partner rats that pulled, or it experienced no help by three different partner rats that did not pull (Figure 1B). Subsequently, we tested the focal rat's propensity to help another unfamiliar partner by recording the number of pulls it performed in a given period (Figure 1C). One day after the experiment, we noted the pulling rate of each focal rat when alone in the experimental cage to check for intrinsic differences in pulling frequency (Figure 1D). The situation was equivalent to the experiment, where the rat could move the platform into the cage by pulling but was unable to reach the reward.

## Results

**Figure 2:**
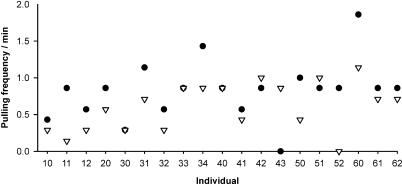
Experience Influences Cooperation Rats pulled more often after help experience (filled dots) than after receiving no help (open triangles). On average the rats' propensity to pull was 21% higher in the test treatment. Numbers with the same first digit denote individuals from the same family (e.g., 10, 11, and 12 were siblings).

Test rats that recently experienced help pulled more often than when they had not experienced help (Figure 2). The pulling frequency was on average 21% higher in the helper treatment than in the nonhelper treatment (median = 0.86 pulls/min compared to 0.71 pulls/min; *Z* = −2.462; *n* = 19; and *p* = 0.014). The median interval between placing an oat flake on the platform and pulling by the rat was shorter after cooperative experience than after receiving no help. Rats with previous experience of help pulled on average four times earlier (medians: helper treatment, 6 s; nonhelper treatment, 24 s; *Z* = −2.486; *n* = 17; and *p* = 0.013; this difference is similar when the two rats that did not pull in one of the two treatments are included in the analysis, assuming an infinite pulling delay; *Z* = 2.133; *n* = 19; and *p* = 0.033). However, the latency of the very first pull that the rats performed in the test situation did not differ significantly between both treatments (medians: helper treatment, 13 s; nonhelper treatment, 34 s; *Z* = −0.853; *n* = 17; and *p* = 0.39). The baseline pulling frequency when alone in the cage was lower (median = 0.29 pulls/min) than in the test phases of the helper treatment (*p* < 0.01) and nonhelper treatments (*p* = 0.017), respectively, and it did not differ between treatments (medians = 0.29 and 0.29; *n* = 10 + 9; *U* = 43.5; and *p* = 0.84). Therefore, the intrinsic tendency to pull was not influenced by the experimental treatments.

## Discussion

Our results show that prior social experience changes the propensity of rats to cooperate, irrespective of the identity of the partner. After experiencing cooperation a rat is more helpful towards a new partner than after receiving no help. This indicates that reciprocal cooperation in iterated encounters is not necessarily based on specific knowledge about the partner, but that any prior experience of cooperation can be used. It is worth noting that pulling the stick was a cooperative act to the rats; they pulled very little when alone in the cage, and their intrinsic pulling frequencies did not differ after being subjected to the helper or nonhelper treatments.

Which alternative learning mechanisms might explain the behavior of rats in our experiment? Instrumental conditioning can be excluded as a mechanism to explain the observed behavior, because the test rat was not rewarded for her own behavior during the experience phase. Classical conditioning is unlikely, as in the nonhelper treatment focal rats also received the same amount of food. Also, both forms of conditioning are not supported by the data, because the intrinsic tendency to pull as measured at the end of the experiment was clearly not influenced by the experimental treatments. We can exclude that the focal rats in the nonhelper treatment learned that the platform would not work, because the nonhelper partner rats had not been trained in the pulling task and therefore did not even try to manipulate the stick or to pull. Also, the very first pulling latency of test rats did not differ between treatments. For the latter reason it is also unlikely that the differing performance was caused by “forgetting” how to perform the task in the nonhelper situation, when observations of a pulling rat were not as recent as in the helper treatment. Social learning is also implausible to be responsible for the different behavior of rats in both treatments; experience and test phases were performed on different days, so social facilitation was not involved. True imitation is unlikely, because (1) the focal rat was able to perform the required behavior already long before the experimental test in both the helper and nonhelper treatments, (2) the focal rat did not perform the behavior herself right after observing the partner rat in the experience phase, but only about 24 h later, and (3) imitation could hitherto not be demonstrated in rats despite intensive study [31].

Generalized reciprocity is hence the only hypothesis fully consistent with our results; the rats helped an unknown conspecific more readily because they received help before, even if from another anonymous partner. This is compatible with an “anonymous generous tit-for-tat”-like strategy, which was shown to establish cooperation in small groups [20]. To our knowledge, this is the first evidence for generalized reciprocity in nonhuman animals. The existence of this form of cooperation does not necessarily imply, however, that other selective forces are not at work in rats. In a follow-up study we tested whether the propensity to cooperate would be increased further when Norway rats interacted with a known partner who had helped them before [32]. As expected, this direct reciprocity caused even higher levels of cooperation than generalized reciprocity, i.e., a rat was 50.7% more likely to help a conspecific who had helped her before than an unknown rat after experiencing cooperation with anonymous partners. This is compatible with a “hierarchical information hypothesis” assuming that specific information about the helping propensity of a partner is used if available, but if not, anonymous social experience is used when deciding whether to cooperate or not [32], i.e., cooperation may ensue also when specific information is limited or costly to be obtained. A similar mechanism might operate in humans [29]. Theoretical models showed that the existence of direct reciprocity in a population will induce the evolution of generalized reciprocity [22], entailing much higher levels of cooperation overall. It is worth noting that despite the fact that direct reciprocity also operates in Norway rats, the results of this study cannot be accounted for by direct reciprocity in connection with errors in identifying individuals; the same individuals were tested in both experimental situations, so recognition errors cannot have biased the results in one direction.

Generalized reciprocity is functionally related to the winner and loser effects, where anonymous social experience also influences behavior in subsequent interactions (in this case agonistic behavior [33–35]). On the proximate level, physiological and neurological mechanisms causing winner/loser effects and generalized reciprocity might be similar [36]. It has been demonstrated experimentally that primates and rats exposed to socio-positive or socio-negative experience show significant hormonal changes [37,38]. These may critically affect the tendency to cooperate. Recently, oxytocin was shown to influence human prosocial behavior [39,40], and it might also mediate positive social interactions in nonhuman animals [41]. A neurological study of human cooperative behavior showed that in women playing the prisoner's dilemma game, mutual cooperation was associated with consistent activation in brain areas linked with reward processing [42], e.g., the anteroventral striatum. When electrodes are placed in the striatum of rats, the animals will repeatedly press a bar to stimulate the electrodes [43]. Rilling and coworkers [42] suggested that the activation of these brain areas might positively reinforce reciprocal altruism.

Our experiment revealed that cooperative behavior of Norway rats is influenced by anonymous social experience, despite their ability to distinguish individuals and their tendency to help particularly those who have helped them before [32]. We believe that this result may affect future studies of cooperation in two important ways. First, empirical data suggesting the potential operation of direct reciprocity may sometimes be interpreted more parsimoniously in terms of generalized reciprocity. So far, adequate controls to differentiate between direct and generalized reciprocity are missing in empirical studies of reciprocal altruism [44–46], except for a study on chimpanzees demonstrating partner-specific exchange of altruistic acts [47] and our study on rats [32]. Second, theoretical approaches attempting to explain cooperation in an evolutionary context should account for the potential involvement of generalized reciprocity.

## Materials and Methods

### Subjects.

The rats were bred from eight pairs of wild-type rats (Animal Physiology Department, University of Groningen, Netherlands) and housed with same-sex littermates in groups of three to seven in cages (80 cm × 50 cm × 37.5 cm). Female groups could not interact with each other between cages because of the arrangement of cages. The housing room had an average temperature of 22 °C and a 12:12 h light:dark cycle with lights on at 20:00 hours. Food (conventional rat pellets) and water was provided ad libitum. Rats are predominantly nocturnal, and thus we performed our experiments during the dark phase in the morning hours.

### Pre-experimental training.

Only female rats were used in the experiment. The training of the rats in the operant cooperative task (which is similar to the task used in [44,46]) consisted of two steps. First, a single rat learned to pull a stick fixed to a baited platform to move it into the cage and reach the reward (one oat flake). All rats learned to pull the stick in this situation within the first two trials of 10 min each. Second, each rat learned to pull alternately with a littermate, providing access to food for each other (Figure 1A). For this, the two rats were placed in a cage that was separated into two compartments by a wire mesh. Only one rat had access to the stick and the opportunity to move the baited platform into the cage. The pulling rat had no access to the reward, only its partner did. In a subsequent session the roles were exchanged. Initially the partners pulled shortly after each other (i.e., one partner had to pull four times, then, the roles were exchanged immediately). The interval between the exchanges of roles was gradually increased to two days over a period of eight weeks. During this training phase each rat had 35 sessions in which she was in the role of the donor and 35 sessions in which she was in the role of the receiver, and she only interacted with one specific littermate. All rats pulled in this cooperative situation. The pulling rate was significantly higher when the partner was present than when the second compartment was empty (medians [Wilcoxon-Test]: alone, 0.5; partner present, 0.8; *p* < 0.001; *n* = 20), indicating that the propensity to pull was socially influenced (see [48]). Thus the rats learned to cooperate by pulling the stick and to reciprocate with a specific partner. In our experiment we used this learned instrumental cooperative behavior to test the influence of social experience on cooperation in rats.

### Experiment.

In the experiment, only rats were paired that were unfamiliar with each other (i.e., had not interacted before) and came from different cages (i.e., were not closely related). The focal rats were first exposed to a situation where they either received help (test treatment) or not (control treatment) from different partners to get food (Figure 1B). In this experience phase the focal rats themselves could not pull for their respective partners. All 19 focal rats were exposed to both treatments in a random sequence. In the test treatment, on five successive days the rats received help from three different partners that pulled and moved the reward within reach of the focal rat (i.e., two partner rats were used twice). Each focal rat had only one session per day. The nine different helping partners had been trained in alternated pulling and were randomly assigned to the test rats. As an incentive each helping partner had been rewarded for pulling shortly before the test rat was put into the experimental cage. The session continued until the partner had pulled eight times, which was achieved on average within seven minutes. In the control treatment, the rats were paired with three different partners that did not pull. The nine nonhelping partners had not been trained in pulling, and the platform was mechanically prevented from moving towards the cage. In any other respect they did not differ from the helping partners, including familiarity with the experimental cage. Each partner was randomly assigned to the control rat. Again, the duration of a trial was seven minutes, and each focal rat had one session per day on five consecutive days. During each control session the experimenter also baited the tray eight times with one oat flake on the side of the focal rat. After each session the partner rat was removed first, and the focal rat received the eight oat flakes on the tray. To test for potential differences in behavior of partner rats between the test and control treatments during the experience phase, we compared the behavior of the nine trained and nine nontrained partners when paired with the same focal rat. The analysis revealed that there was neither a difference in the partner rats' social interactions with the focal rat (*p* = 0.86 and *Z* = −0.178), nor in their general activity (*p* = 0.26 and *Z* = −1.125), nor in the time spent in the quadrant in which they had access to the stick (*p* = 0.77 and *Z* = −0.296) (Wilcoxon-Tests). Therefore, to the best of our knowledge the only difference for the focal rats between treatments was experiencing help or not.

On day six, each focal rat was paired with a new partner and consequently was in the role of the potential helper (Figure 1C). The number of pulls performed by the rat was noted during a period of seven minutes. After four days, we repeated the experimental procedure by switching the experience treatment given to the focal rats. The partner rats providing the opposite experience were again new to the focal rats, whereas in the subsequent test helping behavior of each focal rat was tested with the same individuals as in the preceding treatment. Again, the focal rat herself did not pull in the entire experience phase. Observations were conducted in a blind fashion such that the experimenter did not know which focal rat was in the trial. The experimenter recorded the interactions on a monitor while sitting behind a sliding door. A new oat flake was placed on the platform ten seconds after each pulling event (i.e., after the partner rat usually had consumed the food). One day after the experiment, we compared the pulling rate of ten focal rats that had received help in the second part of the experiment with the pulling rate of nine focal rats that had not received help recently, when alone in the experimental cage (Figure 1D). The rats could move the platform into the cage by pulling but were unable to reach the reward.

### Statistics.

Data were analyzed with nonparametric statistics using the software package SPSS 11.0 (SPSS, http://www.spss.com). We compared individuals across treatments using two-tailed Wilcoxon matched-pairs signed-ranks tests. Bonferroni correction was applied to account for multiple testing (pulling frequency and latency), thus reducing the significance level to α′ = 0.025. For analyzing differences in pulling frequency between treatment groups after the experiment we used the Mann-Whitney U-Test.
